# Dimethylarginine Dimethylaminohydrolase-1 Transgenic Mice Are Not Protected from Ischemic Stroke

**DOI:** 10.1371/journal.pone.0007337

**Published:** 2009-10-07

**Authors:** Frank Leypoldt, Chi-Un Choe, Mathias Gelderblom, Eike-Christin von Leitner, Dorothee Atzler, Edzard Schwedhelm, Christian Gerloff, Karsten Sydow, Rainer H. Böger, Tim Magnus

**Affiliations:** 1 Department of Neurology, University Medical Center Hamburg-Eppendorf, Hamburg, Germany; 2 Department of Cardiology, Hamburg University Heart Center, Hamburg, Germany; 3 Clinical Pharmacology Unit, Institute of Experimental and Clinical Pharmacology and Toxicology, University Medical Center Hamburg-Eppendorf, Hamburg, Germany; Julius-Maximilians-Universität Würzburg, Germany

## Abstract

**Background:**

Methylated arginines are endogenous analogues of L-arginine, the substrate for nitric oxide (NO) synthase. Asymmetric dimethylarginine (ADMA) interferes with NO formation, causing endothelial dysfunction. ADMA is a predictor of cardiovascular events and mortality in humans. It is eliminated primarily by enzymatic activity of dimethylarginine dimethylaminohydrolase (DDAH).

**Methodology/Principal Findings:**

We investigated whether human DDAH-1 (hDDAH-1) transgenicity protects from ischemic tissue damage in temporary middle cerebral artery occlusion (tMCAO) in mice. Infarct sizes did not significantly differ between hDDAH-1 transgenic (TG) mice and wild-type littermates (WT). As expected, ADMA plasma concentrations were significantly decreased, cerebral hDDAH expression and protein significantly increased in transgenic animals. Interestingly, neither brain tissue DDAH activity nor ADMA concentrations were different between TG and WT mice. In contrast, muscular DDAH activity was generally lower than in brain but significantly increased in TG mice.

**Conclusion/Significance:**

Our study demonstrates that hDDAH-1 transgenic mice are not protected from ischemic cerebral tissue damage in tMCAO. This lack of protection is due to high basal cerebral DDAH activity, which is not further increasable by transgenic overexpression of DDAH.

## Introduction

Ischemic stroke is a devastating disease representing the second leading cause of death in the western world and the leading cause of disability in adults. Asymmetric dimethylarginine (ADMA) is an inhibitor of nitric oxide (NO) synthesis and has been shown to be associated with endothelial dysfunction (for review see [1]) whereas symmetric dimethylarginine (SDMA) does not inhibit NO synthases. Accordingly, ADMA impaired cerebral perfusion in rats [2], and infusion of ADMA to healthy human subjects increased arterial stiffness and decreased cerebral perfusion [3]. Elevated ADMA plasma levels are predictive for mortality and future cardiovascular events in humans [4], [5]. ADMA was also reported to be a weak independent risk factor for stroke and TIA [6] and is positively associated with internal carotid atherosclerosis [7]. It is eliminated primarily by the enzymatic activity of dimethylarginine dimethylaminohydrolase (DDAH) [8].

The aim of our study was to investigate whether mice overexpressing an hDDAH-1 transgene and exhibiting decreased plasma concentrations of ADMA [9], are protected from experimental cerebral tissue damage due to ischemic stroke.

## Methods

All animal experiments have been conducted according to relevant national and international guidelines (German Animal Welfare Act) and have been approved by the local Animal Care and Use Committee (Behörde für Soziales, Familie, Gesundheit und Verbraucherschutz - Lebensmittelsicherheit und Veterinärwesen - 26/07). Temporary middle cerebral artery occlusion (tMCAO) was achieved as previously described (male TG and WT littermates, 20–25 g, 10–12 weeks) [10]. Briefly, mice were anesthetized (isoflurane 1–2% v/v oxygen) and analgesized (buprenorphine 0.03 mg/kg b.w. i.p. every 12 h for 24 h). tMCAO was achieved by using the intraluminal filament method (6–0 nylon) for one hour. In the sham group, arteries were visualized but not ligated. Exemplary mice were monitored using transcranial temporal laser Doppler and every mouse was scored on a scale from 0–5 (0 no deficit, 1 preferential turning, 2 circling, 3 longitudinal rolling, 4 no movement, 5 death) after reawakening and every day until sacrifice. Mice were sacrificed 2 days after reperfusion using isoflurane and decapitation. Blood samples were obtained at time of sacrifice. Only mice with a score greater or equal than one after reawakening were included. Of 16 WT and 14 TG mice, 14 respectively 11 mice were included. Brains were harvested, cut into 1 mm standardized slices (Braintree Scientific, 1 mm) and vital stained using 2% (w/v) 2,3,5-triphenyl-2H-tetrazolium chloride (TTC) in phosphate buffer. Slices were scanned on a flat bed scanner, infarct volume determined by blinded examiners using NIH ImageJ. Infarcts were categorized as strictly striatal or territorial (striatal plus cortical) and analyzed separately. Edema corrected infarct size was calculated (Volume Infarct*Volume contralesional hemisphere/Volume ipsilesional hemisphere) and statistics (two-sided T-test, Graph Pad Prism) performed. For india ink vascular staining, WT and TG animals were anesthetized (isoflurane) and perfused with PBS followed by 4% paraformaldehyde and india ink (50% india ink, 5% gelatine in PBS). After refrigerating (4°C) overnight, mice were dissected under a binocular microscope and visualized using a digital camera (Dino Lite Pro).

Western blots were performed according to standard protocols (rabbit polyclonal antibodies against DDAH1 (Eurogentec, Köln, Gemany) and against β-tubulin (Abcam, Cambridge, MA). Circulating plasma levels of ADMA, SDMA, and L-arginine were determined using a validated high-throughput liquid chromatography - tandem mass spectrometry (LC-MS/MS) assay. DDAH activity was quantified in brain and skeletal muscle homogenates by measuring the degradation of [^2^H_6_]-ADMA (n = 6–8) [11]. Real time PCR was performed from brain tissue (WT n = 8, TG n = 7) according to manufacturers' protocols using standard conditions (7900 Fast system, Applied Biosystems, Darmstadt, Germany, Hs00201707 m1 for hDDAH1, Mm01319453 m1 for DDAH1, and Mm00516768 m1 for DDAH2). Significance was tested by two-sided T-test (Graph Pad Prism).

## Results

**Figure 1:**
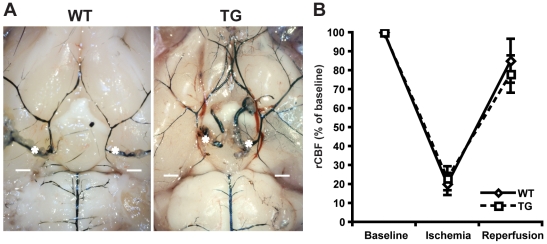
A Cerebral vascular anatomy is not influenced by genotype. Representative cerebral vasculature india ink staining in WT and TG mice. * Branching of internal carotid artery, white dash: partially hidden location of anastomosis of internal carotid and basilar territories. Less intense staining of vessels in the depicted WT animal is due to small differences in india ink perfusion pressure. No systematic difference in vascular architecture was observed (WT n = 4, TG n = 4). B Transtemporal laser doppler analysis of relative cortical blood flow dynamics after insertion of filament shows no difference between WT and TG mice. Baseline was defined as pre-ischemia cerebral blood flow and defined as 100%. WT n = 3, TG n = 3.

**Figure 2:**
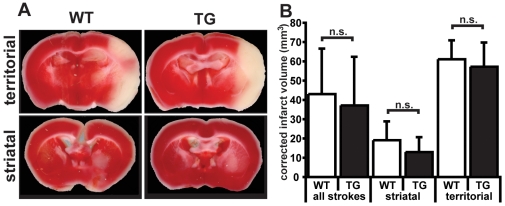
hDDAH-1 transgenic mice are not protected from experimental stroke. A Representative vital staining (TTC) of WT and TG mice brain sections B Infarcted area in mm^3^ (edema corrected, WT n = 14, TG n = 11, striatal WT n = 6 TG n = 5, territorial WT n = 8 TG n = 6). Error bars represent standard deviation. N.s. not significant.

The analysis was 80% powered to see a 20% reduction in infarct size. Direct effects of genotype on development of vasculature could severely bias our ischemia model and needed to be ruled out. We neither found differences in vascular anatomy nor blood flow reduction/reperfusion between TG and WT animals (Figure 1). However, we did not observe significantly different infarct sizes in our model. Edema corrected infarct sizes were in WT 43 mm^3^ (n = 13, 95% CI 24–61) and in TG 37 mm^3^ (n = 11, 95% CI 17–57) (p = 0.49, Fig. 2). Because striatal infarcts were slightly more common in WT animals (6/8) than in TG mice (5/8), we analyzed striatal and territorial infarcts separately. This also did not lead to significant differences. Infarct sizes were 19 mm^3^ (n = 6, 95% CI 11–27) for striatal and 61 mm^3^ (n = 8, CI 54–68) for territorial infarcts in WT mice versus 13 mm^3^ (n = 5, CI 6–20) for striatal and 59 mm^3^ (n = 6, CI 47–67) for territorial infarcts in TG animals (p = 0.27 for striatal and p = 0.53 for territorial infarcts) (Fig. 2).

**Figure 3:**
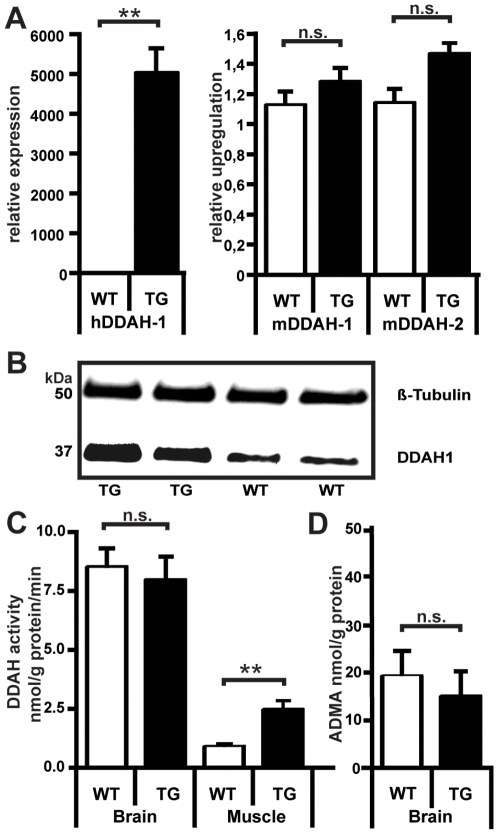
Cerebral DDAH activity is not increased in hDDAH-1 transgenic mice despite a significant difference in hDDAH-1 expression and translation. Muscular DDAH activity is increased but generally lower than cerebral DDAH activity. A Quantitative PCR. Relative expression in TG vs. WT mice. (n = 7–8) B Western blot, brain homogenate. Beta-tubulin and DDAH-1 antibodies (n = 3) C Cerebral and muscular DDAH activity. Brain and muscle homogenate (n = 6–8) D ADMA concentration. Brain homogenate (n = 6–8). Error bars represent standard deviation. WT wildtype; TG hDDAH-1 transgenic animals, n.s. not significant, ** p<0.01.

To explain the lack of protection, we examined ADMA levels as well as expression and enzymatic activity of endogenous and transgenic DDAH. As expected, circulating ADMA plasma concentration was decreased by 40% in TG animals as compared to WT (p<0.01, Table 1). For SDMA and L-arginine, no significant differences were seen (Table 1). The L-arginine:ADMA ratio was significantly higher in TG mice due to lower ADMA levels, while the L-arginine:SDMA ratio did not differ significantly (Table 1). Endogenous cerebral murine DDAH-1 and -2 mRNA expression was not significantly different, while transgenic human hDDAH-1 mRNA levels in brain were only detectable in TG mice (Fig. 3A). Accordingly, DDAH-1 protein was increased in cerebral tissue of TG mice (Fig. 3B).

**Table 1:** Mean plasma levels with 95% confidence intervalls in brackets.

	WT	TG	p
ADMA µmol/l	0.72 (0.60–0.83)	0.43 (0.34–0.53)	**<0.01**
SDMA µmol/l	0.18 (0.16–0.21)	0.17 (0.12–0.23)	0.73
L-Arginine µmol/l	72.7 (50.2–95.1)	95.0 (61.6–128.5)	0.30
Arg:ADMA	104.1 (76.5–131.6)	231.3 (166.0–296.5)	**<0.01**
Arg:SDMA	403.5 (290.7–516.3)	662.0 (451.2–872.8)	0.05

Statistics by two-sided T-test.

Surprisingly, in spite of increased transcription and translation of transgenic protein in the brain, we did not find a significant difference in brain DDAH activity (WT 8.5±0.8 vs. TG, 7.9±1.0 nmol/g protein/min; Fig. 3C) or cerebral tissue ADMA levels (WT 19.1±2.2 vs. TG, 14.8±2.0 nmol/g protein; Fig. 3D) between transgenic animals and littermates. In contrast in muscle tissue, muscular DDAH activity was significantly increased in TG animals (WT 0.8±0.1 vs. TG, 2.3±0.4 nmol/g protein/min; Fig. 3C). However, littermate and transgenic DDAH activities of skeletal muscle were only 10% and 30% of brain DDAH activity, respectively.

## Discussion

Previously, higher vasomotor capacities in medium- to small-sized arteries and improved perfusion in hDDAH-1 transgenic mice were demonstrated [2], [9]. However, in our study we observed no effect of transgenicity on cerebral infarct size. As the human β-actin promoter was used to construct the transgene [12], ubiquitous expression of mRNA and enhanced DDAH activity in large arteries was anticipated. In accordance with this, we found expression of hDDAH-1 both on the mRNA and protein level in brains of TG mice. Nevertheless, we show here that DDAH activity in the brain of TG mice was not different from WT animals and concomitantly cerebral ADMA tissue levels remained unchanged.

Interestingly, in organs where elevated DDAH activity had been found in transgenic mice [12], [13] protection from ischemia was noticeable. For example, in models of hind limb and myocardial ischemia increased angiogenesis and reduced damage were observed [14], [15]. Fittingly, we showed increased muscular DDAH activity in transgenic animals but with a much lower activity level compared to the brain. With regard to the transgenic model it seems that no further increase in DDAH activity can be achieved in the brain explaining our observed lack of protection from stroke.

Recent evidence suggests that DDAH-1 overexpression protects from endothelial dysfunction in cerebral arterioles [9]. Therefore, local endothelial effects of increased DDAH-expression have been postulated. The lack of a protective effect of the DDAH-1 transgene in our model with significantly different ADMA plasma levels points to a minor relevance of these observations for infarct size. Correspondingly, epidemiologic data shows ADMA to be only a minor risk factor for stroke in humans [6]. Currently, the influence of DDAH transgenicity on non-vascular, rather than vascular mechanisms contributing to secondary cerebral damage can not be answered. Further work to elucidate non-vascular effects of DDAH-1 is needed.

In summary, our findings suggest that hDDAH-1 overexpression and concurrent reduction in plasma ADMA concentrations do not change the outcome of stroke. This is likely due to the inability of the transgene to further increase already high background activity of DDAH in the brain.
